# A report of a new species and new record of *Cadlina* (Nudibranchia, Cadlinidae) from South Korea

**DOI:** 10.3897/zookeys.996.54602

**Published:** 2020-11-24

**Authors:** Thinh Dinh Do, Dae-Wui Jung, Hyun-Jong Kil, Chang-Bae Kim

**Affiliations:** 1 Department of Biotechnology, Sangmyung University, Seoul 03016, South Korea Sangmyung University Seoul South Korea; 2 Korea Marine-Bio Lab, Daejeon 34130, South Korea Korea Marine-Bio Lab Daejeon South Korea; 3 Genetic Resources Information Center, National Institute of Biological Resources, Incheon 22689, South Korea National Institute of Biological Resources Incheon South Korea

**Keywords:** *Cadlina koreana* sp. nov., description, northwestern Pacific region, morphology, phylogeny

## Abstract

Of the four species in the genus *Cadlina* present in the northwestern Pacific region, *C. japonica* has been the only species recorded from South Korea. For the purpose of investigating *Cadlina* in Korean waters, specimens were collected from the Korean East Sea (Sea of Japan) by scuba diving. The radula and morphology of these specimens were examined by stereoscopic and scanning electron microscopy. Based on morphology, three species were identified in Korean waters, including the new species, *Cadlina koreana***sp. nov.**, *C. umiushi* (first record in South Korea), and *C. japonica*. *Cadlina koreana***sp. nov.** somewhat resembles *C. umiushi* but differs in both its morphology as well as the structure of its radula. The background color of *Cadlina koreana***sp. nov.** is translucent white, tubercles on the dorsum are opaque white and the yellow marginal band is absent. The radular formula of *Cadlina koreana***sp. nov.** is 57 × 23.1.23 with a rectangular rachidian tooth. In addition, mitochondrial cytochrome c subunit 1 (COI), 16S ribosomal RNA (16S rRNA), and nuclear 28S ribosomal RNA (28S rRNA) gene sequences were generated and used for analysis of Automatic Barcode Gap Discovery (ABGD) and reconstruction of the phylogenetic tree. Morphological distinction and genetic analyses confirm that three *Cadlina* species are present in Korean waters of which *Cadlina koreana* is a new species.

## Introduction

*Cadlina* Bergh, 1879 is a genus of slow-moving and flattened dorid nudibranchs (Korshunova et al. 2020). *Cadlina* species are reported as common dorid nudibranchs in northern temperate waters but become remarkably scarcer in tropical regions (Schrödl 2000). Recently, the genus was extensively reviewed by Korshunova et al. (2020). In that study, the authors described four new species and re-described *C. umiushi* Korshunova et al., 2015 and *C. kamchatica* Korshunova et al., 2015. Their findings increase the understanding of *Cadlina* species in the northern seas, especially in the northwestern Pacific region. To date, there have been four *Cadlina* species recorded in the northwestern Pacific region: *C. japonica* Baba, 1937, *C. kamchatica*, *C. umiushi*, and *C. paninae*Korshunova et al., 2020. Of these, only *C. japonica* was previously recorded from South Korea by Choe and Lee (1994). Potentially, there are more *Cadlina* species present in Korean waters awaiting discovery.

Members of the nudibranch genus *Cadlina* generally have similar body shapes and coloration so it is a difficult task to distinguish them based on their morphology (Korshunova et al. 2020). DNA barcoding is widely reported as an effective tool for both the identification of known species and the discovery of new species (Hebert et al. 2003). Because of the difficulty in identifying *Cadlina* species from morphology only, molecular markers have been analyzed to improve the accuracy of species discrimination (Korshunova et al. 2020). Mitochondrial and nuclear markers such as COI, 16S rRNA, and 28S rRNA genes are often selected for analysis. In previous studies of nudibranchs, these markers were used in combination with a morphological examination to discover new species and separate species complexes (Lindsay and Valdés 2016; Korshunova et al. 2020).

This study aimed to investigate *Cadlina* species in Korean waters. For this purpose, eight specimens were collected for species identification. In addition, fragments of COI, 16S rRNA, and 28S rRNA genes from these specimens were sequenced and analyzed to compare with the morphological examinations.

## Materials and methods

### Sample collection and morphological examination

*Cadlina* species were collected from the Korean East Sea (Sea of Japan) by scuba diving. Upon collection, specimens were preserved in 10% neutral buffered formalin for morphological examination. In addition, small sample of tissue from the foot was stored in 95% ethanol for DNA extraction. Sample collection data and depositories are presented in Suppl. material 1: Table S1. A stereoscopic microscope (Nikon SMZ800N) was used to examine the specimens. The buccal mass was extracted under a stereo microscope for radula extraction. The buccal mass was placed in 10% KOH for two days at room temperature to dissolve muscle. The radula was then carefully removed from the solution and placed in deionized water for 20 minutes to remove excess KOH. The radulae were examined under a JEOL JSM-6390LV scanning electron microscope (Jeol Inc., USA). The reproductive systems were dissected under a stereoscopic microscope and drawn with a camera lucida. Morphological comparison and species descriptions were prepared following previous guidelines (Schrödl 2000; Chichvarkhin 2016; Korshunova et al. 2020).

### Molecular analysis

Total DNA was extracted from the foot of each specimen using E.Z.N.A. Mollusc DNA Kit (Omega Bio-tek, USA). The quality and concentration of the extracted DNA were checked using a MaestroNano spectrophotometer (Maestrogen, Taiwan). Polymerase chain reaction (PCR) analysis was performed for two mitochondrial markers (COI and 16S rRNA) and one nuclear marker (28S rRNA). The primer set for each marker is listed in Table 1.

**Table 1.** Primer sets of COI and 16S rRNA and 28S rRNA genes used in this study.

Primer	Gene	Sequence (5’-3’) genes	Annealing temperature	Reference
LCO1490	COI	GGTCAACAAATCATAAAGATATTGG	45°	Folmer et al. 1994
HCO2198		TAAACTTCAGGGTGACCAAAAAATCA		
16Sar-L	16S rRNA	CGCCTGTTTATCAAAAACAT	48°	Palumbi 1996
16S R		CCGRTYTGAACTCAGCTCACG		Puslednik and Serb 2008
28S C1	28S rRNA	ACCCGCTGAATTTAAGCAT	48°	Hassouna et al. 1984
28S C2		TGAACTCTCTCTTCAAAGTTCTTTTC		Le et al. 1993

The 20 μl PCR reaction mixture contained 10 μl of 2X TOPsimple DyeMIX-Tenuto (Enzynomics, South Korea), 1 μl of each primer (10 pmoles/μl), 100 ng of DNA, and distilled water. The amplification protocol was as follows: initial denaturation at 95 °C for 5 minutes, followed by 35 cycles of denaturation at 95 °C for 45 seconds, variable annealing temperature for each primer set as listed in Table 1 for 45 seconds, extension at 72 °C for 1 minute, and final elongation at 72 °C for 5 minutes. The PCR products were checked by electrophoresis in 1% agarose gels in 1× TAE buffer. Sequencing was performed by an ABI 3730 DNA Analyzer (Applied Biosystems, USA).

Consensus sequences were generated from the forward and reverse sequences with Geneious software version 9.1.8 (Kearse et al. 2012). The obtained sequences were submitted to GenBank and the sequence accession numbers are listed in Suppl. material 1: Table S1. The sequences were compared with sequences in GenBank using the BLAST tool to search for related species. Additionally, sequences of the genus *Cadlina* were obtained from GenBank for Automatic Barcode Gap Discovery (ABGD) analysis and phylogenetic reconstruction. The ABGD webtool (https://bioinfo.mnhn.fr/abi/public/abgd/abgdweb.html) was applied to delineate putative species based on COI and 16S rRNA sequences (Puillandre et al. 2012). The distance matrices for COI and 16S rRNA were built in MEGA X software using the Kimura 2-parameter model (Kumar et al. 2018). The default settings used for analysis were Pmin = 0.001, Pmax = 0.1, Steps = 10, X = 1.5, Nb bins = 20. All three different distance models are available from the ABGD webtool: Simple Distance, Jukes-Cantor (JC69), and Kimura (K80) TS/TV were tested (Puillandre et al. 2012).

Phylogenetic reconstruction of *Cadlina* species was conducted based on the concatenation of three markers (COI, 16S rRNA, and 28S rRNA) or two markers (COI and 16S rRNA) because there were no 28S rRNA sequences for some species. Two species of the genus *Aldisa*, *A. sanguinea* and *A. smaragdina*, in the family Cadlinidae were used as the outgroup. Before concatenation, each marker was aligned using the ClustalW method in MEGA X software (Kumar et al. 2018) and poorly aligned regions were trimmed by GBlocks 0.91b (Castresana 2000). The Akaike Information Criterion in jModelTest 2.1.10 was used to search for the best model for phylogenetic tree reconstruction (Darriba et al. 2012). The phylogenetic trees were reconstructed using both the Maximum Likelihood (ML) and Bayesian Inference (BI) methods. The ML phylogenetic tree was constructed using the GTR+G+I model with 1000 bootstrap replicates in MEGA X software (Kumar et al. 2018). The BI tree was reconstructed in MrBayes ver. 3.2.7a with two runs for 10 million generations and a sampling interval of 1000 generations (Ronquist et al. 2012).

## Results

### Morphological results

#### Cadlina koreana sp. nov.

Taxon classificationAnimaliaNudibranchiaCadlinidae

A5DEA3ED-F668-5D0B-8508-7C0E6D448F98

http://zoobank.org/BDAF5119-92FB-499A-BD02-63EB7077ABED

Figures 1 , 4A

##### Type material.

***Holotype*.** NIBRIV0000865970; South Korea, Gangwon-do, Goseong-gun, Jugwang-myeon, Munamjin-ri; 38°18'14.75"N, 128°34'1.05"E; collected on 02 June 2013 (COI GenBank number: MT420429). ***Paratype*.** NIBRIV0000865971; South Korea, Gangwon-do, Goseong-gun, Jugwang-myeon, Munamjin-ri; 38°18'14.75"N, 128°34'1.05"E; collected on 02 June 2013 (COI GenBank number: MT420430).

##### Other material.

Voucher: SMU00051; South Korea, Gangwon-do, Goseong-gun, Jugwang-myeon, Munamjin-ri; 38°18'14.75"N, 128°34'1.05"E; collected on 02 June 2013 (COI GenBank number: MT420431).

##### Diagnosis.

**Figure 1.**
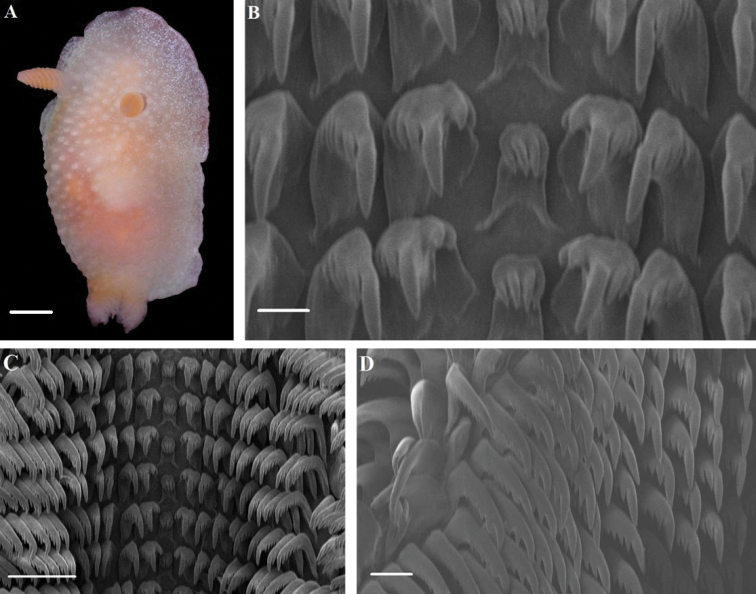
*Cadlina koreana***A** body; Radula **B** rachidian teeth **C** central and lateral region **D** outer lateral region. Scale bars: 2 mm (**A**); 10 μm (**B**); 50 μm (**C**); 20 μm (**D**).

Ground color translucent white (Fig. 1A). Rhinophores and gills opaque white to translucent yellow. Entire dorsum covered by small rounded tubercles with white coloration. Radula formula 57 × 23.1.23. Rachidian tooth rectangular with four main sharp cusps (Fig. 1B). Innermost lateral teeth massive, wide base; cusp strong, slightly curved; two inner denticles and three to four outer denticles (Fig. 1C). Outer lateral teeth hamate, well-defined denticles (Fig. 1D).

##### Description.

Body elongated ovate; body lengths 10.3 mm (holotype), 14 mm (paratype), and 9 mm (additional specimen). Ground color translucent white (Fig. 1A). Dorsum broad in front and posteriorly. Mantle broad and wider than foot; thin at the edge. Numerous white specks present at mantle edge, lacking a yellow marginal band. Dorsum covered with numerous small white tubercles. Rhinophores opaque white to translucent yellow; clavus lamellate; cylindrical stalk smooth. Rhinophoral sheath smooth. Gills opaque white to yellow, six multi-pinnate branchial leaves, retractable into gill cavity. Gill sheath bears small nodules. Oral veil forms triangular, lateral sides. Foot anteriorly rounded and thickened. Radula formula: 57 × 23.1.23. Rachidian tooth rectangular with four sharp denticles; two central denticles slightly longer than two lateral denticles (Fig. 1B). Innermost lateral tooth massive with one large, slightly curved cusp; two short, inner denticles; and three to four outer denticles. Second lateral tooth with one cusp, no inner denticle and four or five outer denticles. Middle lateral tooth hamate with one large cusp and up to seven denticles (Fig. 1C). Outer lateral teeth hamate, comb-shaped with 5–7 clearly visible denticles (Fig. 1D). Reproductive system triaulic (Fig. 4A). Ampulla moderate, convoluted, and connects with female gland and prostate. Prostate long and narrow. Seminal vesicle slightly more swollen than prostate. Vas deferens narrow, smooth, and distinct. Penis armed with spines. Vagina relatively narrow and connects with bursa copulatrix. Bursa copulatrix ovate and ca. 1.5 × larger than receptaculum seminis. Uterine duct short and narrow, bifurcates into female gland and receptaculum seminis.

##### Remarks.

A comparison of *Cadlina* species recorded in the northwestern Pacific region is presented in Table 2. *Cadlina koreana* sp. nov. is most similar externally to *C. paninae*, differing in color variation of the rhinophores and gills as well as the structure of the radula. In *C. paninae*, the color of the rhinophores and gills are opaque white while in *Cadlina koreana*, color of the rhinophores can vary from opaque white to translucent yellow and that of the gills can vary from opaque white to yellow. *Cadlina koreana* also has fewer rows and fewer denticles on both the rachidian tooth and its lateral tooth compared to those of *C. paninae*. In addition, the rachidian tooth of *C. paninae* is often bifurcated at the tips while the rachidian tooth of *Cadlina koreana* is not bifurcated at the tips.

**Table 2.** Morphological comparison among *Cadlina* species in the northwestern Pacific region.

Species	Locality	Size	Morphology	Radular formula	Rachidian tooth	First lateral teeth	Mid-lateral teeth	Outer lateral teeth	Ampulla	Vas deferens	Vagina	Bursa copulatrix and receptaculum seminis	Source of information
*Cadlina koreana*	Munamjin-ri, South Korea	9–14 mm	Translucent white; dorsum covered with small white tubercles; small white specs present on mantle edge. No yellow marginal band	57 × 23.1.23	Rectangular, hook-shaped, 2 longer central denticles, and 2 shorter lateral denticles	1 cusp, 2 short inner denticles, and 3–4 outer denticles.	Hamate, comb-shaped, up to 7 denticles	Hamate, comb-shaped, 5–7 denticles.	Moderate and convoluted	Long and narrow	Relatively long and narrow	Ovate and ca. 1.5 × larger than receptaculum seminis	This study
*Cadlina umiushi*	Munamjin-ri, South Korea	8–9 mm	White background; numerous small yellow tubercles; yellow marginal band	55 x 16.1.16	Trapezoid, hook-shaped, 2 central denticles, and 2 lateral denticles	1 cusp, 2 inner denticles, and 3 outer denticles	Hamate, rather comb-shaped, 6–8 distinct outer denticles.	Hamate to straight, up to 10 inconspicuous denticles	Long and convoluted	Relatively short	Relatively short and broad	Ovate and ca. 2 × larger than receptaculum seminis	This study
*Cadlina japonica*	Munamjin-ri and Yeonji-ri, South Korea	48–55 mm	Yellowish with dark brown patches; small scattered yellow spots; yellow marginal band	88 x 71.1.71	Elongate, 2–4 lobe-like denticles	1 bigger cusp, 3–4 inner denticles, and 4–6 outer denticles	Hook-shaped, no inner denticle and 3–5 outer denticles	Hook-shaped, bearing up to 6 denticles	Moderate and convoluted	Long, narrow and distinct	Relatively short and narrow	Almost rectangular in shape, ca. 5 × larger than receptaculum seminis	This study
*Cadlina kamchatica*	Kamchatka, Starichkov Island, Russia	37 mm	Creamy to dark yellow/light brown; small, low rounded yellow tubercles	82 × 35.1.35	Moderately high, trapezoid, 5–6 denticles, 2 middle usually larger than outer ones	1 cusp, 4–6 large inner denticles, 5–6 distinct outer denticles	Hamate, comb-shaped, up to 17 distinct outer denticles only	Hamate reduced, up to 19 sharp denticles	Long and convoluted	Relatively short	Long and narrow	Pear-shaped, 2 × larger than receptaculum seminis	Korshunova et al. (2020)
*Cadlina paninae*	Matua Islands, Middle Kurile Islands, Russia	29 mm	Opaque whitish, sometimes with some yellowish shadow; low indistinct tubercles	90 × 38.1.38	Low rectangular, 3–5 distinct cusps, often bifurcated at tips	1 cusp, 2–3 inner denticles and 3–4 outer denticles	Elongate hook-shaped, up to 20 comb-shaped denticles	Hook-shaped, up to 20 comb-shaped denticles	Relatively short and slightly convoluted	Long and narrow	Long and narrow	Ovate, 1.5 × larger than receptaculum seminis	Korshunova et al. (2020)

The external morphology of *Cadlina koreana* is relatively similar to *C. umiushi*, which also has small-sized tubercles on the dorsum. However, clear differences between these two species can be observed by comparing their coloration. The color pattern of *Cadlina koreana* is white without yellow tubercles or a yellow marginal band. In contrast, *C. umiushi* is semi-transparent white with yellow tubercles and a yellow marginal band. The rachidian tooth of *Cadlina koreana* is rectangular while it is trapezoid in *C. umiushi*. The inner denticles of the first lateral tooth of *C. umiushi* are half the length of the tooth body, but in *Cadlina koreana* it is less than half the length of the tooth body. Moreover, the outer lateral teeth of *Cadlina koreana* are hamate with clearly visible denticles. In contrast, the outer lateral teeth of *C. umiushi* are almost straight with inconspicuous denticles.

*Cadlina japonica* is distinguished from *Cadlina koreana* sp. nov. by brownish patches on the dorsum and an elongate rachidian tooth with lobe-like denticles. *Cadlina kamchatica* clearly differs from *Cadlina koreana* by its yellowish body color and the higher number of denticles on the rachidian tooth and lateral tooth. The common *Cadlina* species in the northeastern Pacific, *C. luteomarginata* MacFarland, 1966, differs from *Cadlina koreana* by yellow dots on the dorsum and a yellow rim to the mantle. The other species in this region, *C. flavomaculata* MacFarland, 1905, also has yellow dots on the dorsum that are not present in *Cadlina koreana*. The color of *C. modesta* MacFarland, 1966 is light yellowish to light brown while it is translucent white in *Cadlina koreana*. Compared to *Cadlina koreana*, three *Cadlina* species recently described by Korshunova et al. (2020), *C. klasmalmbergi*, *C. jannanicholsae*, and *C. sylviaearleae* have yellow mantle bands and yellow tubercles. In *Cadlina koreana*, the yellow mantle band is absent and the color of the tubercles is white. The maximum intraspecific distances in *C. koreana* are 0% for the COI marker and 0.23% for the 16S rRNA marker (Suppl. material 1: Table S3). The lowest COI interspecific distance of 5.78% is found between *C. koreana* and *C. umiushi*. The lowest 16S rRNA interspecific distance of 4.56% is found between *C. koreana* and *C. paninae*.

##### Etymology.

The species is named after the country of its type locality.

##### Distribution.

*Cadlina koreana* sp. nov. is currently known only from Munamjin-ri, South Korea.

#### Cadlina umiushi

Taxon classificationAnimaliaNudibranchiaCadlinidae

Korshunova, Picton, Sanamyan & Martynov, 2015

7639246B-3F13-5A01-BA57-3DCB6CE6DEEB

Figures 2 , 4B

Cadlina umiushi Korshunova, Picton, Sanamyan & Martynov, 2015 in Martynov et al. 2015: 65, fig. 1; Korshunova et al. 2020: 15, 29, figs 7, 15B.
Cadlina olgae Chichvarkhin, 2016: 12–14, fig. 4.

##### Material examined.

One individual, voucher NIBRIV0000865972; South Korea, Gangwon-do, Goseong-gun, Jugwang-myeon, Munamjin-ri; 38°18'14.75"N, 128°34'1.05"E; collected on 02 June 2013 (COI GenBank number: MT420435). One individual, voucher SMU00060; South Korea, Gangwon-do, Goseong-gun, Jugwang-myeon, Munamjin-ri; 38°18'14.75"N, 128°34'1.05"E; collected on 02 June 2013 (COI GenBank number: MT420436).

##### Description.

**Figure 2.**
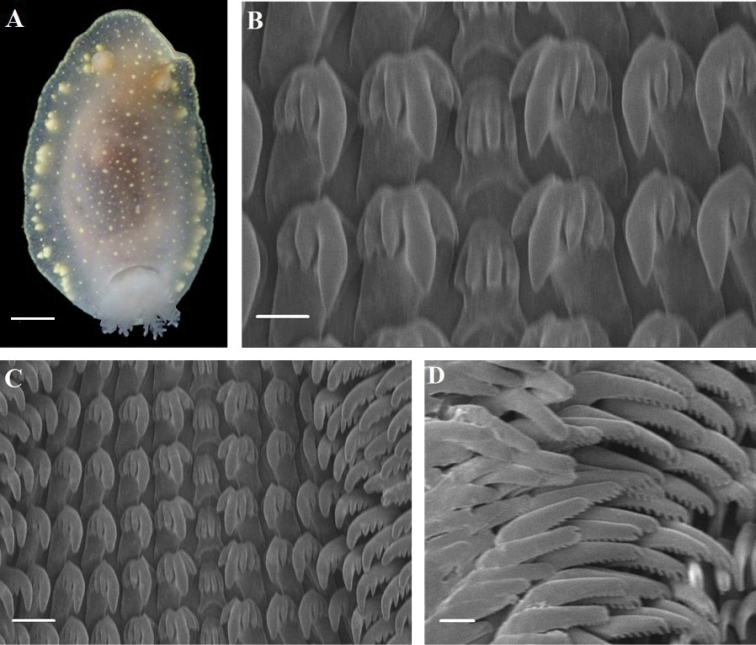
*Cadlina umiushi***A** body; Radula **B** rachidian teeth **C** central and lateral region **D** outer lateral region. Scale bars: 2 mm (**A**); 10 μm (**B**); 50 μm (**C**); 20 μm (**D**).

Body ovate, 8 mm and 9 mm long. Living specimens with a translucent white dorsum (Fig. 2A). Small yellow glands are present on both sides of the dorsum margin. Thin yellow marginal band present. Dorsum broad, rounded anteriorly and posteriorly. Small yellow tubercles cover the entire dorsum. Rhinophores long and broad. Six multipinnate gills connected by a membrane into circle around anus. Gills retractable into gill cavity. Foot broad, anteriorly thickened. Radula formula: 55 × 16.1.16. Rachidian tooth moderately high, trapezoid, and bearing four denticles (Fig. 2B). Innermost lateral tooth massive with one cusp, two inner denticles, and three outer denticles. Second lateral tooth with one cusp, no inner denticles and three outer denticles. Mid-lateral tooth hamate, 6–8 distinct outer denticles (Fig. 2C). Outer lateral tooth almost straight, denticles small, inconspicuous (Fig. 2D). Reproductive system triaulic (Fig. 4B). Ampulla long, wide, convoluted. Prostate moderate in length and wide, transiting to vas deferens. Vas deferens relatively short. Penis armed with spines. Vagina relatively short and broad, connecting with oval bursa copulatrix. Bursa copulatrix ca. 2 × larger than receptaculum seminis. Uterine duct short and narrow, connecting from female gland mass to base of ovate receptaculum seminis.

##### Remarks.

*Cadlina umiushi* was first described in Martynov et al. (2015) from the holotype specimen collected in Peter the Great Bay, Russia. *Cadlina olgae* Chichvarkhin, 2016, described from specimens collected in south of Rudnaya Bay, Russia is considered a junior synonym of *C. umiushi* by Korshunova et al. (2020). This study records the presence of *C. umiushi* in Korean waters for the first time. Even though there were slight differences in the number of denticles of the rachidian and the first lateral tooth, and in the ampulla compartments of specimens collected from South Korea compared to the specimens collected from Russia, other morphological characteristics are similar. It should be noted that this difference is also observed between specimens in Russia collected by Chichvarkhin (2016) and Korshunova et al. (2020). The differences could be explained by the geographical distribution or maybe a different stage of development. The maximum intraspecific distances in *C. umiushi* are 1.56% for the COI marker and 1.37% for the 16S rRNA marker (Suppl. material 1: Table S3). The lowest COI interspecific distance of 4.33% is found between *C. umiushi* and *C. laevis* (Linnaeus, 1767). The lowest 16S rRNA interspecific distance of 1.37% is found between *C. umiushi* and *C. kamchatica*.

##### Distribution.

Northern part of Sea of Japan (Russia) to Munamjin-ri (South Korea).

#### Cadlina japonica

Taxon classificationAnimaliaNudibranchiaCadlinidae

Baba, 1937

EE96E4F0-FDF2-57EC-8F76-BD051F5EADE6

Figures 3 , 4C

Cadlina japonica Baba, 1937: 76–78, fig. 1; Baba 1949: 57, pl. XXI, figs 75–77, text fig. 67; Choe and Lee 1994: 362, fig. 2; Nakano 2018: 275; Korshunova et al. 2020: 36–39, figs 11, 12.

##### Material examined.

Two individuals, vouchers: NIBRIV0000865973 and NIBRIV0000865974; South Korea, Gangwon-do, Goseong-gun, Jugwang-myeon, Munamjin-ri; 38°18'14.75"N, 128°34'1.05"E; collected on 02 June 2013 and 20 July 2019 (COI GenBank numbers: MT420432 and MT420433). One individual, voucher NIBRIV0000865975; South Korea, Gyeongsangbuk-do, Uljin-gun, Uljin-eup, Yeonji-ri; 37°00'0.59"N, 129°26'1.89"E; collected on 25 August 2011 (COI GenBank number: MT420434).

##### Description.

**Figure 3.**
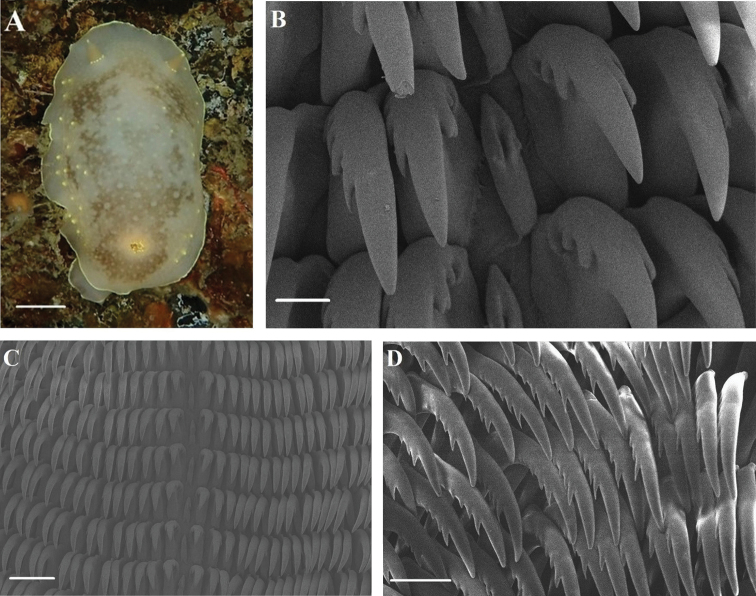
*Cadlina japonica***A** body; Radula **B** rachidian teeth **C** central and lateral region **D** outer lateral region. Scale bars: 1 cm (**A**); 20 μm (**B**); 100 μm (**C**); 50 μm (**D**).

**Figure 4.**
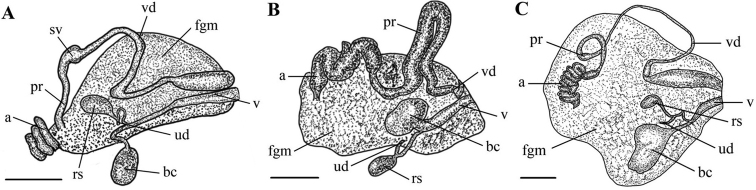
Reproductive systems of *Cadlina* species **A***Cadlina koreana* sp. nov. **B***Cadlina umiushi***C***Cadlina japonica*. Abbreviations: a, ampulla; bc, bursa copulatrix; fgm, female gland mass; rs, receptaculum seminis; pr, prostate; sv, seminal vesicle; ud, uterine duct; v, vaginal duct; vd, vas deferens. Scale bars: 0.5 mm (**A, B**); 2 mm (**C**).

Size up to 55 mm long. Live specimens commonly opaque white with a yellowish ground color and several dark brownish patches present on the dorsum (Fig. 3A). Rhinophores yellowish like the ground color with bright yellow tips. Rhinophoral sheath bears small tubercles and yellow dots. Six multipinnate, translucent white gills with yellow tips. Yellow dots present irregularly on the dorsum, but often concentrated near the mantle margin. Continuous yellow band present on mantel edge. Oral tentacles short and triangular. Foot broad, anteriorly thickened to form a double edge. Radula formula: 88 × 71.1.71. Rachidian tooth elongate and bears 2–4 distinct lobe-like cusps (Fig. 3B). Innermost lateral tooth hamate with a relatively narrow base and short, strong cusp; three or four inner denticles and 4–6 outer denticles (Fig. 3B). Middle lateral teeth hook-shaped, no inner denticle and 3–5 outer denticles (Fig. 3C). Outer lateral teeth bear up to six denticles (Fig. 3D). Reproductive system triaulic (Fig. 4C). Ampulla is moderate and convoluted. Prostate long and narrow. Vas deferens long, narrow, convoluted. Penial spines absent. Vagina relatively short, narrow and connects with bursa copulatrix. Bursa copulatrix almost rectangular in shape, ca. 5 × larger than receptaculum seminis. Uterine duct short and narrow.

##### Remarks.

*Cadlina japonica* was first described by Baba (1937). Recently, this species was thoroughly reviewed by Korshunova et al. (2020). Morphologically, the *C. japonica* specimens collected in this study are consistent with *C. japonica* described in previous studies (Baba 1937; Choe and Lee 1994; Korshunova et al. 2020). *Cadlina japonica* is completely distinguishable from other species of the genus by several characteristics. Irregular brownish patches are present on the mantle, but these patches are absent on several small individuals. Its rachidian tooth is elongate and its first lateral tooth is hamate. In addition, compared to other *Cadlina* species in the region, *C. japonica* is large, with the sample in this study measuring up to 55 mm. The maximum intraspecific distances in *C. japonica* are 0.78% for the COI marker and 0.23% for the 16S rRNA marker (Suppl. material 1: Table S3). The lowest COI interspecific distance of 7.97% is found between *C. japonica* and *C. jannanicholsae*Korshunova et al., 2020. The lowest 16S rRNA interspecific distance of 1.87% is found between *C. japonica* and *C. klasmalmbergi*.

##### Distribution.

Southern Hokkaido to southern Honshu (Japan) and East Sea, South Korea (Sea of Japan).

### Molecular analyses

Analyses of the three molecular markers also demonstrated differences between *Cadlina koreana* sp. nov. and other *Cadlina* species recorded in GenBank. The BLAST results showed that *C. umiushi* is the closest species to *Cadlina koreana* with 93.8% and 95.3% similarity in the COI and 16S rRNA genes, respectively. The number of taxonomic groups based on ABGD analysis for COI varied from 11 to 13, depending on the intraspecific divergence prior (p) value (Suppl. material 1: Table S4). In the 11-groups partition, all input species including three species in this study and species in Suppl. material 1: Table S3 were recovered, and each species corresponded to a distinct group. In the 12- and 13-groups partition, *Cadlina koreana* sequences were always clustered together. The same pattern was also observed for *C. japonica*. Meanwhile, *C. umiushi* sequences were partitioned into two or three groups, depending on the p value. ABGD analysis for 16S rRNA revealed 9–5 groups (Suppl. material 1: Table S5). Similar to the COI analysis, in the 11-group partition, all input species were recovered. *Cadlina koreana* sequences always formed a distinct group for all partitions. Similarly, *C. japonica* sequences also formed a group, except in the 15-group partition. In this partition, the p value was minimum and two groups of *C. japonica* were observed. In the 9-group partition, *C. umiushi* was grouped with *C. kamchatica* and *C. paninae*. When the p value decreased, the total number of groups increased to 15 and *C. umiushi* sequences were divided into three groups.

**Figure 5.**
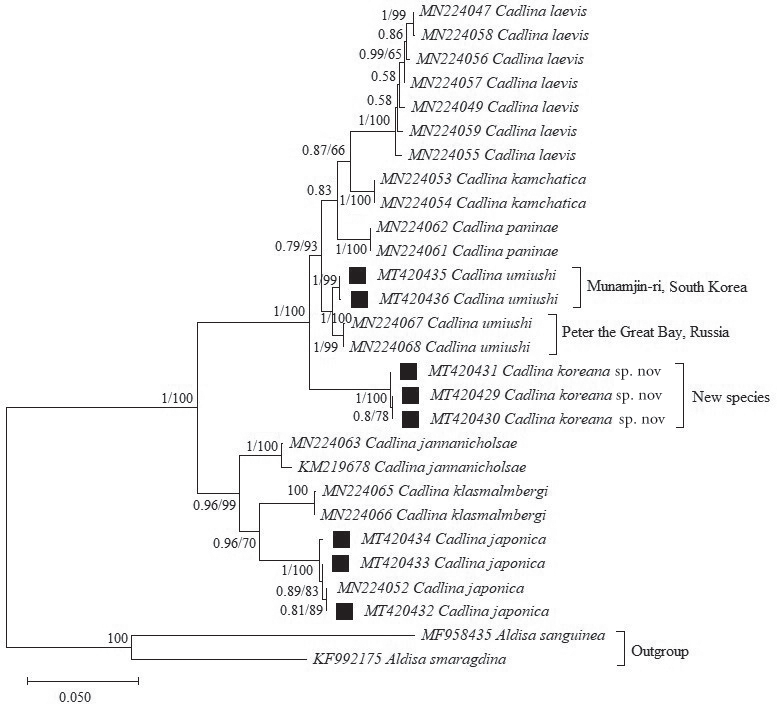
Phylogenetic tree based on concatenation of COI, 16S rRNA, and 28S rRNA markers. Sequences generated in this study are marked with back squares; the remaining sequences were obtained from GenBank. Accession numbers of COI sequences appear in front of species names to identify specific specimens as in Suppl. material 1: Tables S1, S2. The tree was constructed using the Maximum Likelihood method with 1000 bootstrap replicates in MEGA X software and Bayesian Inference in MrBayes software. *Aldisa sanguinea* and *A. smaragdina* were used as the outgroup. Numbers at each node indicated bootstrap (right) and posterior probability (left) values. The values > 50 (BS) and 0.5 (PP) are provided.

A phylogenetic tree of three concatenated markers (COI, 16S rRNA, and 28S rRNA) was reconstructed to determine the positions of the three species of *Cadlina* found in South Korea (Fig. 5) while the phylogenetic tree of two concatenated markers (COI and 16S rRNA) was reconstructed to resolve the relationship of as many species in the genus *Cadlina* as possible (Suppl. material 2: Fig. S1). The ML and BI trees based on the three concatenated markers show a similar pattern (Fig. 5). The phylogenetic tree of the three concatenated markers indicates that *Cadlina koreana* specimens form an independent branch that is sister to a group that includes *C. umiushi*, *C. kamchatica*, *C. paninae*, and *C. laevis*. Moreover, the *C. umiushi* specimens were clustered together and formed two groups, a Russian group from Peter the Great Bay and a South Korean group from Munamjin-ri. Meanwhile, *C. japonica* was clustered with sequences from the same species available in GenBank (Fig. 5). The ML and BI trees of two concatenated markers (COI and 16S rRNA) had slightly different topology patterns (Suppl. material 2: Fig. S1A, S1B). However, both trees showed a separated position of *Cadlina koreana*. In the tree from three concatenated markers, the *C. umiushi* specimens from south of Rudnaya, Russia were added for analysis due to the availability of COI and 16S rRNA sequences. As a result, *C. umiushi* formed three branches according to geographical distributions, including two Russian branches (Peter the Great Bay and south of Rudnaya Bay) and one South Korean branch (Munamjin-ri). Both trees of two concatenated markers and three concatenated markers showed that *C. japonica* specimens were clustered with sequences from GenBank, and no clear separated groups were observed (Fig. 5; Suppl. material 2: Fig. S1).

## Discussion

Species of the genus *Cadlina* are widely distributed in the northern temperate regions. *Cadlina japonica*, the first species of this genus reported from the northwestern Pacific region (Baba 1937), was described based on specimens collected in Japan; this species was also recorded in South Korea (Baba 1937; Choe and Lee 1994). Compared to other the *Cadlina* species, *C. japonica* is easily recognized by its distinct brownish patches on the mantle and distinct morphology of the rachidian and lateral teeth; the specimens examined in this work are similar to previous descriptions. The second and third species found in the region, *C. umiushi* and *C. kamchatica*, were described by Martynov et al. (2015). The latest species, and most similar to our new species, *C. paninae*, was recently described by Korshunova et al. (2020). With unambiguous evidence from morphological and molecular analyses, the present study identified three species in Korean waters: *C. japonica*, *C. umiushi*, and a new species named *Cadlina koreana*.

*Cadlina koreana* is the fifth species recorded in the northwestern Pacific region. The new species can be differentiated from all previously described species by a combination of morphological and molecular markers. Similar to most *Cadlina* species, the ground color of *Cadlina koreana* is white. However, the distinct characteristics of *Cadlina koreana* are the absence of both the yellow tubercles on the dorsum and a yellow marginal band, two features present in most *Cadlina* species found in the northern Pacific (Korshunova et al. 2020). The observations of radulae by SEM also support the distinction of *Cadlina koreana*: the shape of the rachidian and lateral teeth as well as the radula formula distinguishes it from the other species.

Moreover, the presence of *C. umiushi* in Korean waters is described for the first time. The morphology of *C. umiushi* collected in the present study resembled that of other specimens described in previous studies (Chichvarkhin 2016; Korshunova et al. 2020). Similar to those reports, the dorsum of *C. umiushi* in this study was broad with small yellow tubercles. Also, there was a yellow mantle band on the specimens. The radula of specimens collected from Munamjin-ri, South Korea showed almost perfect resemblance with those of specimens described by Chichvarkhin (2016) and Korshunova et al. (2020), except for slight differences in the radular formulae and the numbers of denticles in the rachidian and first lateral teeth (Table 2). The radula formula (55 × 16.1.16) in this study was closer to the specimens from Chichvarkhin (2016) (55–60 × 13.1.13) than the specimen from Korshunova et al. (2020) (70 × 30.1.30). For the rachidian tooth of the radula, both Chichvarkhin (2016) and Korshunova et al. (2020) reported five or six denticles while there were four denticles in the specimens collected from South Korea. For the first lateral tooth, the specimens in this study included two inner denticles, a distinct cusp, and three outer denticles that were closest to the specimens reported by Korshunova et al. (2020) with two or three inner denticles, a distinct cusp, and 4–6 outer denticles. Also, the morphology of the reproductive system of the specimens collected from the three sites was similar except for the ampulla: even though all specimens showed long and convoluted ampullae, the specimens from Munamjin-ri and south of Rudnaya Bay had two folds, while several compartments were seen in the specimens from Peter the Great Bay (Chichvarkhin 2016; Korshunova et al. 2020).

It is challenging to identify *Cadlina* species based on morphology because of similar characteristics and morphological conservatism. Molecular markers are well known as a useful tool to support the identification of this group (Korshunova et al. 2020). In this study, three molecular markers COI, 16S rRNA, and 28S rRNA were used together with morphological examination. Our molecular analysis confirmed the findings of our morphological study: *Cadlina koreana* sp. nov. and *C. japonica* are distinct species based on ABGD analyses. For both markers, *C. umiushi* sequences were partitioned into a distinct group at a specific p value. For the COI marker, *C. umiushi* sequences were not grouped with any other species. All COI sequences of *C. umiushi* were grouped together or partitioned into two or three groups when the p value decreased. For the 16S rRNA marker, *C. umiushi* sequences can be partitioned into up to three groups, depending on the p value. When the p value was high, all *C. umiushi* sequences formed a group with *C. kamchatica* and *C. paninae*. This finding showed a high intraspecific distance within *C. umiushi* and low interspecific distances between *C. umiushi*, *C. kamchatica* and *C. paninae*. Our results are concordant with a previous study that observed a small gap between the maximum intraspecific distance and the minimum interspecific distance of 16S rRNA sequences of *C. umiushi* (Korshunova et al. 2020), which were 1.18% and 1.41%, respectively. In the present study, when more 16S rRNA sequences from Korean waters were added for estimation, the intraspecific distance within *C. umiushi* became larger (1.37%) and comparable to the distance between *C. umiushi*, *C. kamchatica*, and *C. paninae*. In contrast, even though the COI sequences from our *C. umiushi* specimens were added, *C. umiushi* were not grouped with other *Cadlina* species. In a previous study of aeolid nudibranchs, the COI gene was proven to be better than 16S rRNA gene in resolving the relationship at the species level (Cella et al. 2016).

According to the phylogenetic tree, *Cadlina koreana* sp. nov., *C. japonica*, and *C. umiushi* formed independent clusters. Interestingly, three separate groups of *C. umiushi* were observed that corresponded with the three geographical collection sites. The ABGD and phylogenetic analyses showed some distances within *C. umiushi* among the collection sites. This result was congruent with the morphological examination discussed above and could indicate a possible hidden diversity within this species. It is worth noting that the number of specimens in this study as well as in the surveys of Martynov et al. (2015), Chichvarkhin (2016), and Korshunova et al. (2020) are limited. More *C. umiushi* specimens from different geographical localities must be collected to further elucidate the population structure and speciation of this species.

Based on morphology and analyses of three molecular markers, three *Cadlina* species are identified from South Korea: *Cadlina koreana* sp. nov., *C. umiushi* (a new record for South Korea), and *C. japonica*. These results demonstrate the usefulness of the combination of morphological examination and molecular analyses in species identification, termed integrative taxonomy by Dayrat (2005). This approach should be applied for any future works that deal with the taxonomy of *Cadlina* species. Further studies are necessary to investigate the taxonomy and distribution of *Cadlina* species in the region. This is fundamental to improving our understanding of *Cadlina* diversity and systematics.

## Supplementary Material

XML Treatment for Cadlina koreana

XML Treatment for Cadlina umiushi

XML Treatment for Cadlina japonica

## Supplementary materials

Supplementary material 1 Tables S1–S3 Data type molecular data Explanation note **Table S1.** Collection information and Genbank accession numbers of samples. **Table S2.** Sequences obtained from GenBank used in the present study. **Table S3.** Intraspecific and interspecific distances (%) of *Cadlina* species based on COI and 16S rRNA sequences. Species with multiple sequences available for each marker were targeted for analysis. **Table S4.**ABGD analysis for COI sequences of *Cadlina* species. **Table S5.**ABGD analysis for 16S rRNA sequences of *Cadlina* species. https://binary.pensoft.net/file/478663This dataset is made available under the Open Database License (http://opendatacommons.org/licenses/odbl/1.0/). The Open Database License (ODbL) is a license agreement intended to allow users to freely share, modify, and use this Dataset while maintaining this same freedom for others, provided that the original source and author(s) are credited.Thinh Dinh Do, Dae-Wui Jung, Hyun-Jong Kil, Chang-Bae Kim

Supplementary material 2 Figure S1 Data type molecular data Explanation note Phylogenetic tree based on concatenation of COI and 16S rRNA markers. Sequences generated in this study are marked with black squares; the remaining sequences were obtained from GenBank. Accession numbers of COI sequences appear in front of species names to identify specific specimens listed in Table S1 and Table S2. The tree was constructed using the Maximum Likelihood method with 1000 bootstrap replicates in MEGA X software (**A**) and Bayesian Inference in MrBayes software (**B**). *Aldisa sanguinea* and *A. smaragdina* were used as the outgroup. Numbers at nodes indicate bootstrap and posterior probability values. The values > 50 (BS) and 0.5 (PP) are provided. https://binary.pensoft.net/file/478664This dataset is made available under the Open Database License (http://opendatacommons.org/licenses/odbl/1.0/). The Open Database License (ODbL) is a license agreement intended to allow users to freely share, modify, and use this Dataset while maintaining this same freedom for others, provided that the original source and author(s) are credited.Thinh Dinh Do, Dae-Wui Jung, Hyun-Jong Kil, Chang-Bae Kim
