# Supplementary material for: A report of a new species and new record of Cadlina (Nudibranchia, Cadlinidae) from South Korea
Source: Zookeys. 2020 Nov 24;996:1–18. doi: 10.3897/zookeys.996.54602 (PMC7710686; doi:10.3897/zookeys.996.54602)
Supplement: Supplementary material 1 — Tables S1–S3 [file zookeys-996-001-s001.docx]

**Table S1.** Collection information and GenBank accession numbers of samples.

| **No.** | **Species name** | **Collection site** | **Coordinates** | **Date** | **Storage** | **Specimen catalog** | **GenBank accession number** | | |
| --- | --- | --- | --- | --- | --- | --- | --- | --- | --- |
|  |  |  |  |  |  |  | **COI** | **16S rRNA** | **28S rRNA** |
| 1 | *Cadlina koreana* (holotype) | Munamjin-ri, Jugwang-myeon, Goseong-gun, Gangwon-do, South Korea | 38°18'14.75"N; 128°34'1.05"E | 02.06.2013 | National Institute of Biological Resources, South Korea | NIBRIV0000865970 | MT420429 | MT425042 | MT425034 |
| 2 | *Cadlina koreana* (paratype) | Munamjin-ri, Jugwang-myeon, Goseong-gun, Gangwon-do, South Korea | 38°18'14.75"N; 128°34'1.05"E | 02.06.2013 | National Institute of Biological Resources, South Korea | NIBRIV0000865971 | MT420430 | MT425043 | MT425035 |
| 3 | *Cadlina koreana* | Munamjin-ri, Jugwang-myeon, Goseong-gun, Gangwon-do, South Korea | 38°18'14.75"N; 128°34'1.05"E | 02.06.2013 | Sangmyung University, South Korea | SMU00060 | MT420431 | MT425044 | MT425036 |
| 4 | *Cadlina umiushi* | Munamjin-ri, Jugwang-myeon, Goseong-gun, Gangwon-do, South Korea | 38°18'14.75"N; 128°34'1.05"E | 02.06.2013 | National Institute of Biological Resources, South Korea | NIBRIV0000865972 | MT420435 | MT425048 | MT425040 |
| 5 | *Cadlina umiushi* | Munamjin-ri, Jugwang-myeon, Goseong-gun, Gangwon-do, South Korea | 38°18'14.75"N; 128°34'1.05"E | 02.06.2013 | Sangmyung University, South Korea | SMU00070 | MT420436 | MT425049 | MT425041 |
| 6 | *Cadlina japonica* | Munamjin-ri, Jugwang-myeon, Goseong-gun, Gangwon-do, South Korea | 38°18'14.75"N; 128°34'1.05"E | 02.06.2013 | National Institute of Biological Resources, South Korea | NIBRIV0000865973 | MT420432 | MT425045 | MT425037 |
| 7 | *Cadlina japonica* | Munamjin-ri, Jugwang-myeon, Goseong-gun, Gangwon-do, South Korea | 38°18'14.75"N; 128°34'1.05"E | 20.07.2019 | National Institute of Biological Resources, South Korea | NIBRIV0000865974 | MT420433 | MT425046 | MT425038 |
| 8 | *Cadlina japonica* | Yeonji-ri, Uljin-eup, Uljin-gun, Gyeongsangbuk-do, South Korea | 37°00'0.59"N; 129°26'1.89"E | 25.08.2011 | National Institute of Biological Resources, South Korea | NIBRIV0000865975 | MT420434 | MT425047 | MT425039 |

**Table S2.** Sequences obtained from GenBank used in the present study.

| **No.** | **Species name** | **COI** | **16S rRNA** | **28S rRNA** |
| --- | --- | --- | --- | --- |
| 1 | *Cadlina flavomaculata* | EF534041 | EF535109 | - |
| 2 | *Cadlina flavomaculata* | EU982715 | EU982764 | - |
| 3 | *Cadlina jannanicholsae* | MN224063 | MN224091 | MN224125 |
| 4 | *Cadlina jannanicholsae* | KM219678 | KJ653679 | KP340350 |
| 5 | *Cadlina japonica* | MN224052 | MN224078 | MN224113 |
| 6 | *Cadlina japonica* | MN224051 | MN224077 | - |
| 7 | *Cadlina kamchatica* | MN224053 | MN224079 | MN224114 |
| 8 | *Cadlina kamchatica* | MN224054 | MN224080 | MN224115 |
| 9 | *Cadlina klasmalmbergi* | MN224065 | MN224093 | MN224127 |
| 10 | *Cadlina klasmalmbergi* | MN224066 | MN224094 | MN224128 |
| 11 | *Cadlina laevis* | MN224057 | MN224084 | MN224119 |
| 12 | *Cadlina laevis* | MN224047 | MN224085 | MN224120 |
| 13 | *Cadlina laevis* | MN224058 | MN224086 | MN224121 |
| 14 | *Cadlina laevis* | MN224049 | MN224081 | MN224116 |
| 15 | *Cadlina laevis* | MN224059 | MN224087 | MN224122 |
| 16 | *Cadlina laevis* | MN224055 | MN224082 | MN224117 |
| 17 | *Cadlina laevis* | MN224056 | MN224083 | MN224118 |
| 18 | *Cadlina luarna* | EU982717 | EU982767 | - |
| 19 | *Cadlina luarna* | EU982718 | EU982768 | - |
| 20 | *Cadlina luteomarginata* | EU982721 | EU982771 | - |
| 21 | *Cadlina modesta* | EU982722 | EU982772 | - |
| 22 | *Cadlina modesta* | EU982723 | EU982773 | - |
| 23 | *Cadlina modesta* | MF958437 | MF958310 | - |
| 24 | *Cadlina paninae* | MN224061 | MN224089 | MN224123 |
| 25 | *Cadlina paninae* | MN224062 | MN224090 | MN224124 |
| 26 | *Cadlina paninae* | MN224060 | MN224088 | - |
| 27 | *Cadlina pellucida* | EU982724 | EU982774 | MF958396 |
| 28 | *Cadlina rumia* | EU982725 | EU982775 | - |
| 29 | *Cadlina sparsa* | EU982726 | EU982776 | - |
| 30 | *Cadlina sylviaearleae* | MN224064 | MN224092 | MN224126 |
| 31 | *Cadlina umiushi* | MN224067 | MN224095 | MN224129 |
| 32 | *Cadlina umiushi* | MN224068 | MN224096 | MN224130 |
| 33 | *Cadlina umiushi* | KX610757 | KX938354 | - |
| 34 | *Cadlina umiushi* | KX610756 | KX938355 | - |
| 35 | *Cadlina umiushi* | KX610758 | KX938356 | - |
| Outgroup | *Aldisa sanguinea* | MF958435 | MF958309 | MF958394 |
|  | *Aldisa smaragdina* | KF992175 | KJ022806 | KJ023043 |

-: Sequence is not available.

**Table S3.** Intraspecific and interspecific distances (%) of *Cadlina* species based on COI and 16S rRNA sequences. Species with multiple sequences available for each marker were targeted for analysis.

| **No.** | **Species name** | **Number of specimens** | **COI** | | **16S rRNA** | |
| --- | --- | --- | --- | --- | --- | --- |
|  |  |  | Intraspecific distance (%) | Interspecific distance (%) | Intraspecific distance (%) | Interspecific distance (%) |
| 1 | *Cadlina koreana* | 3 | 0 | 5.78-15.31 | 0-0.23 | 4.56-9.86 |
| 2 | *Cadlina umiushi* | 7 | 0-1.56 | 4.33-15.76 | 0-1.37 | 1.37-9.17 |
| 3 | *Cadlina japonica* | 5 | 0-0.78 | 7.97-16.88 | 0-0.23 | 1.87-9.89 |
| 4 | *Cadlina flavomaculata* | 2 | 0.85 | 9.19-16.88 | 1.37 | 2.05-10.24 |
| 5 | *Cadlina jannanicholsae* | 2 | 0.94 | 7.97-16.56 | 0.23 | 1.64-9.89 |
| 6 | *Cadlina kamchatica* | 2 | 0 | 4.17-15.25 | 0 | 1.37-9.15 |
| 7 | *Cadlina klasmalmbergi* | 2 | 0.16 | 7.97-16.88 | 0 | 1.64-10.45 |
| 8 | *Cadlina laevis* | 7 | 0-1.41 | 4.17-17.03 | 0.22-1.57 | 2.47-10.09 |
| 9 | *Cadlina luarna* | 2 | 0 | 14.69-16.88 | 0 | 7.08-9.89 |
| 10 | *Cadlina modesta* | 3 | 0-0.63 | 9.49-17.03 | 0-0.23 | 2.05-10.48 |
| 11 | *Cadlina paninae* | 3 | 0 | 4.22-16.25 | 0 | 1.60-9.15 |

**Table S4.** ABGD analysis for COI sequences of *Cadlina* species.

| **Partition** | **Taxonomic group** | | | | | |
| --- | --- | --- | --- | --- | --- | --- |
| Prior intraspecific divergence | 0.0129 | 0.077 | 0.0046 | 0.0028 | 0.0017 | 0.001 |
| Initial partition | 7 | 7 | 7 | 7 | 7 | 7 |
| Recursive partition | 11 | 12 | 12 | 13 | 13 | 13 |

**Table S5.** ABGD analysis for 16S rRNA sequences of *Cadlina* species.

| **Partition** | **Taxonomic group** | | | | |
| --- | --- | --- | --- | --- | --- |
| Prior intraspecific divergence | 0.077 | 0.0046 | 0.0028 | 0.0017 | 0.001 |
| Initial partition | 5 | 9 | 9 | 9 | 9 |
| Recursive partition | 9 | 11 | 11 | 15 | 15 |
