# Supplementary material for: A report of a new species and new record of Cadlina (Nudibranchia, Cadlinidae) from South Korea
Source: Zookeys. 2020 Nov 24;996:1–18. doi: 10.3897/zookeys.996.54602 (PMC7710686; doi:10.3897/zookeys.996.54602)
Supplement: Supplementary material 2 — Figure S1 [file zookeys-996-001-s002.docx]

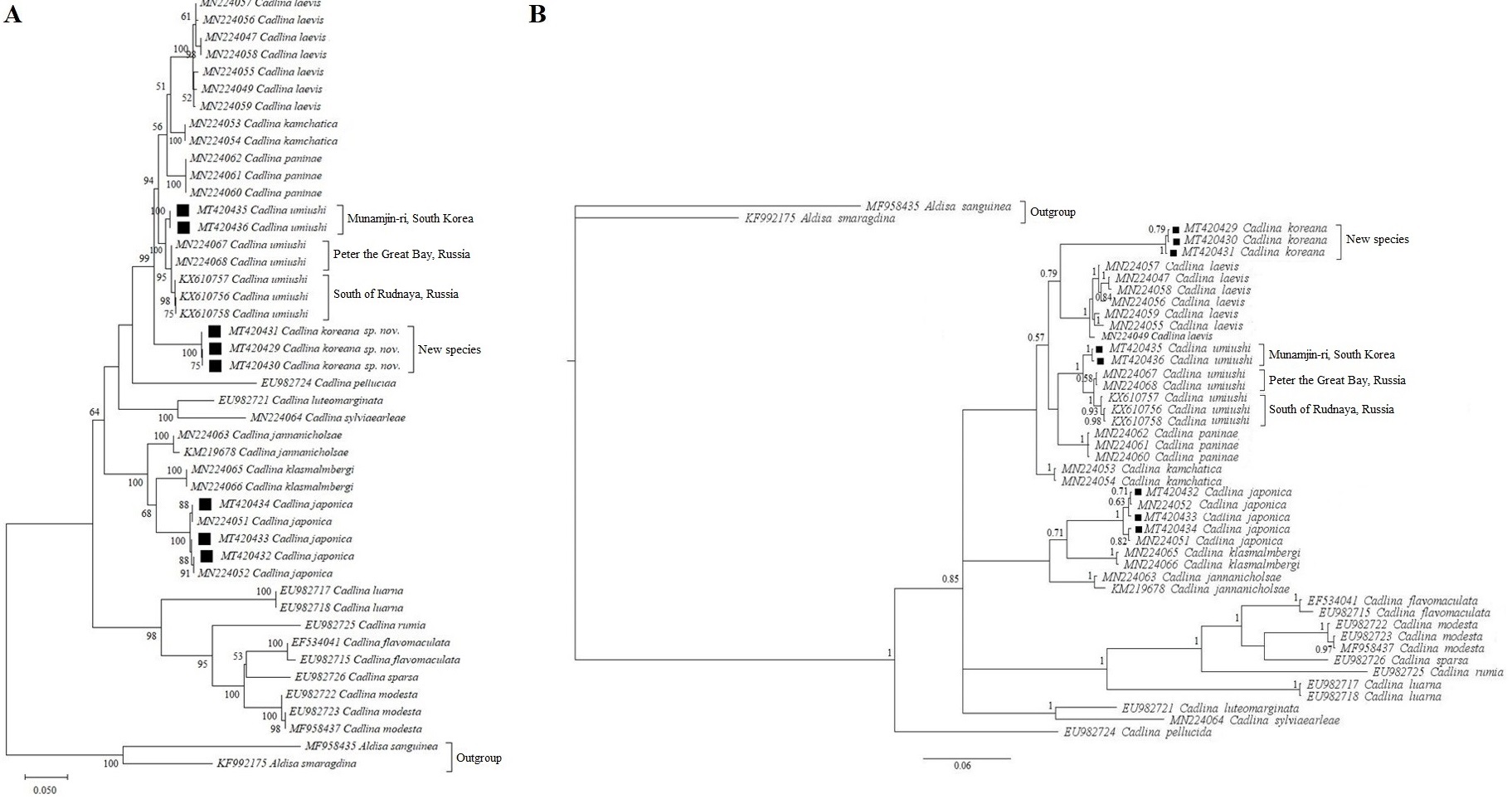


**Figure S1.** Phylogenetic tree based on concatenation of COI and 16S rRNA markers. Sequences generated in this study are marked with black squares; the remaining sequences were obtained from GenBank. Accession numbers of COI sequences appear in front of species names to identify specific specimens listed in Table S1 and Table S2. The tree was constructed using the Maximum Likelihood method with 1000 bootstrap replicates in MEGA X software (**A**) and Bayesian Inference in MrBayes software (**B**). *Aldisa sanguinea* and *A. smaragdina* were used as the outgroup. Numbers at nodes indicate bootstrap and posterior probability values. The values > 50 (BS) and 0.5 (PP) are provided.
